# The small compound, TD-198946, protects against intervertebral degeneration by enhancing glycosaminoglycan synthesis in nucleus pulposus cells

**DOI:** 10.1038/s41598-020-71193-6

**Published:** 2020-08-25

**Authors:** Junichi Kushioka, Takashi Kaito, Ryota Chijimatsu, Rintaro Okada, Hiroyuki Ishiguro, Zeynep Bal, Joe Kodama, Fumiko Yano, Taku Saito, Ung-il Chung, Sakae Tanaka, Hideki Yoshikawa

**Affiliations:** 1 Department of Orthopaedic Surgery, Osaka University Graduate School of Medicine, 2-2 Yamadaoka, Suita, Osaka 565-0871 Japan; 2 Bone and Cartilage Regenerative Medicine, Graduate School of Medicine, The University of Tokyo, 7-3-1 Hongo, Bunkyo-ku, Tokyo, 113-8655 Japan; 3 Sensory and Motor System Medicine, Graduate School of Medicine, The University of Tokyo, 7-3-1 Hongo, Bunkyo-ku, Tokyo, 113-8655 Japan; 4 Center for Disease Biology and Integrative Medicine, The University of Tokyo, 7-3-1 Hongo, Bunkyo-ku, Tokyo, 113-8655 Japan

**Keywords:** Osteoarthritis, Drug development, Translational research, Musculoskeletal system, Cartilage, Skeleton

## Abstract

Degeneration of the nucleus pulposus (NP) might serve as a trigger for intervertebral disc degeneration (IDD). A recent drug screening study revealed that the thienoindazole derivative, TD-198946, is a novel drug for the treatment of osteoarthritis. Because of the environmental and functional similarities between articular cartilage and intervertebral disc, TD-198946 is expected to prevent IDD. Herein, we sought to evaluate the effects of TD-198946 on IDD. TD-198946 enhanced glycosaminoglycan (GAG) production and the related genes in mouse NP cells and human NP cells (hNPCs). Further, Kyoto Encyclopedia of Genes and Genomes pathway analysis using the mRNA sequence of hNPCs suggested that the mechanism of action of TD-198946 primarily occurred via the phosphoinositide 3-kinase (PI3K)/Akt signaling pathway. The Akt inhibitor suppressed the enhancement of GAG production induced by TD-198946. The effects of TD-198946 on IDD at two different time points (immediate treatment model, immediately after the puncture; latent treatment model, 2 weeks after the puncture) were investigated using a mouse tail-disc puncture model. At both time points, TD-198946 prevented a loss in disc height. Histological analysis also demonstrated the preservation of the NP structures. TD-198946 exhibited therapeutic effects on IDD by enhancing GAG production via PI3K/Akt signaling.

## Introduction

Low back pain (LBP) is a common cause of disability and thus acts as a distinct societal and economic burden. Although several factors contribute to the severity or persistence of LBP, intervertebral disc degeneration (IDD) is considered to be one of its leading causes^1–3^. The intervertebral disc consists of a central gel-like nucleus pulposus (NP), an outer annulus fibrosus (AF), and superior and inferior cartilaginous endplates. Among them, NP plays a crucial role in the maintenance of homeostasis of the intervertebral disc via the production of the extracellular matrix (ECM). Aging or mechanical/chemical stress, which disrupts the anabolic and catabolic balance, results in the deterioration of the ECM. Deterioration in NP causes a loss in disc height and structural wear of the intervertebral disc. Further, it promotes IDD^4,5^. Therefore, treating NP degeneration has garnered increased attention.

Previously, regenerative approaches for degenerated NP, including growth factors^6,7^, stem cells^8^, and synthetic biomaterials^9^, have been reported. Although these therapies are promising, their high costs and safety concerns might prevent their widespread use. Small compounds can be employed as an alternative treatment option for common diseases such as IDD and may contribute to lowering cost and safety concerns. Recently, a drug screening study using the COL2-GFP-ATDC5 monitoring system revealed that of the 2,500 natural and synthetic small compounds identified, the thienoindazole derivative, TD-198946, is a novel option for early osteoarthritis (OA). In a mouse OA model, the administration of TD-198946 into the joint space prevented the degeneration of the articular cartilage. Further, TD-198946 was found to induce the production of ECM proteins, such as glycosaminoglycan (GAG) and type 2 collagen from chondrocytes^10^. Both chondrocytes and NP cells are located in a hypoxic avascular environment and secrete ECM components to attain the hydration required to resist compressive loads^11^. Considering the environmental and functional similarities between chondrocytes and NP cells, we hypothesized that TD-198946 could be administered to treat IDD. However, NP and cartilage are significantly different in terms of anatomical structure, GAG composition, and some phenotypic markers^5,12^. Therefore, the effects of TD-198946 on NP cells need to be investigated independently.

In the present study, we aimed to investigate the effects of TD-198946 on NP cells, elucidate its mechanism of action in vitro, and evaluate its treatment potential on IDD using a tail-puncture-induced mouse IDD model.

## Results

### TD-198946 increased GAG synthesis in mouse NP cells (mNPCs)

**Figure 1:**
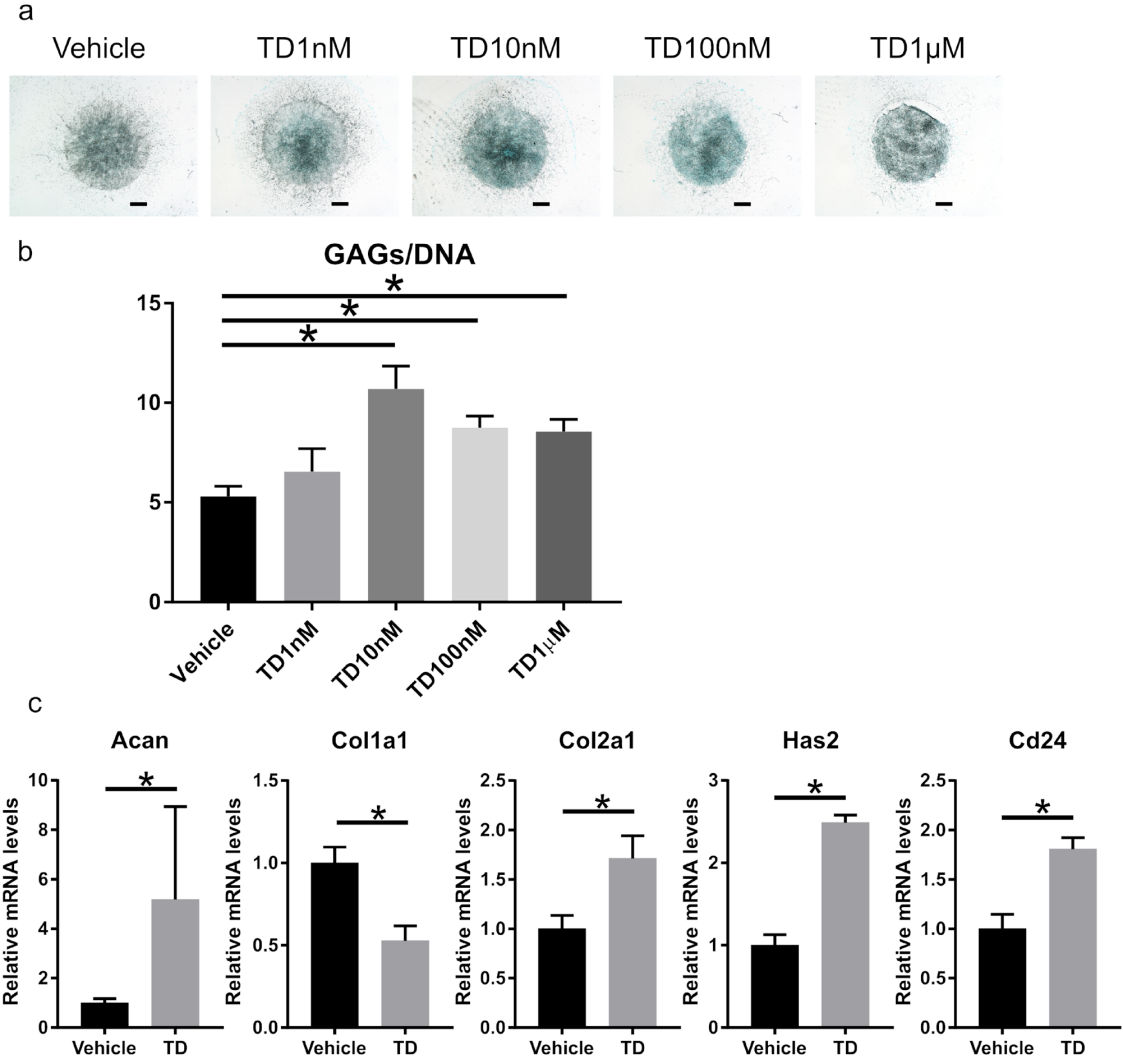
Two-dimensional micromass culture of mouse NP cells. (**a**) Alcian blue staining of mouse NP cells cultured with the vehicle or TD (1 nM to 1 μM) for 7 days (bar = 1,000 μm). (**b**) Sulfated GAG quantification of mouse NP cells cultured with the vehicle or TD (1 nM to 1 μM) for 7 days. Data represent mean ± S.D., n = 4 for each group, *; p < 0.05 by one-way ANOVA followed by the Dunnett test. (**c**) Real-time PCR analysis of mouse NP cells cultured with the vehicle or TD (10 nM) for 7 days. Data represent mean ± S.D., n = 4 for each group, *; p < 0.05, *NS* not significant by Student’s t test. *NP* nucleus pulposus, *TD* TD-198946, *GAG* glycosaminoglycan.

First, we evaluated the effects of TD-198946 on mNPCs in vitro. TD-198946 enhanced the alcian blue staining of mNPCs at a concentration of 10 nM (Fig. 1a). Based on the GAG quantification assay, TD-198946 enhanced GAG synthesis in mNPCs. Further, the most substantial effect was observed at a concentration of 10 nM (Fig. 1b). Gene expression analysis revealed that TD-198946 (10 nM) enhanced the expression of ECM synthesis genes (Acan, Has2, and Col2a1). TD-198946 also enhanced the expression of Cd24, which has been reported as an NP-specific marker^5,13–15^ (Fig. 1c). Thus, the effects of TD-198946 on mNPCs for GAG synthesis were demonstrated.

### TD-198946 enhanced glycosaminoglycan synthesis in human NP cells (hNPCs)

**Figure 2:**
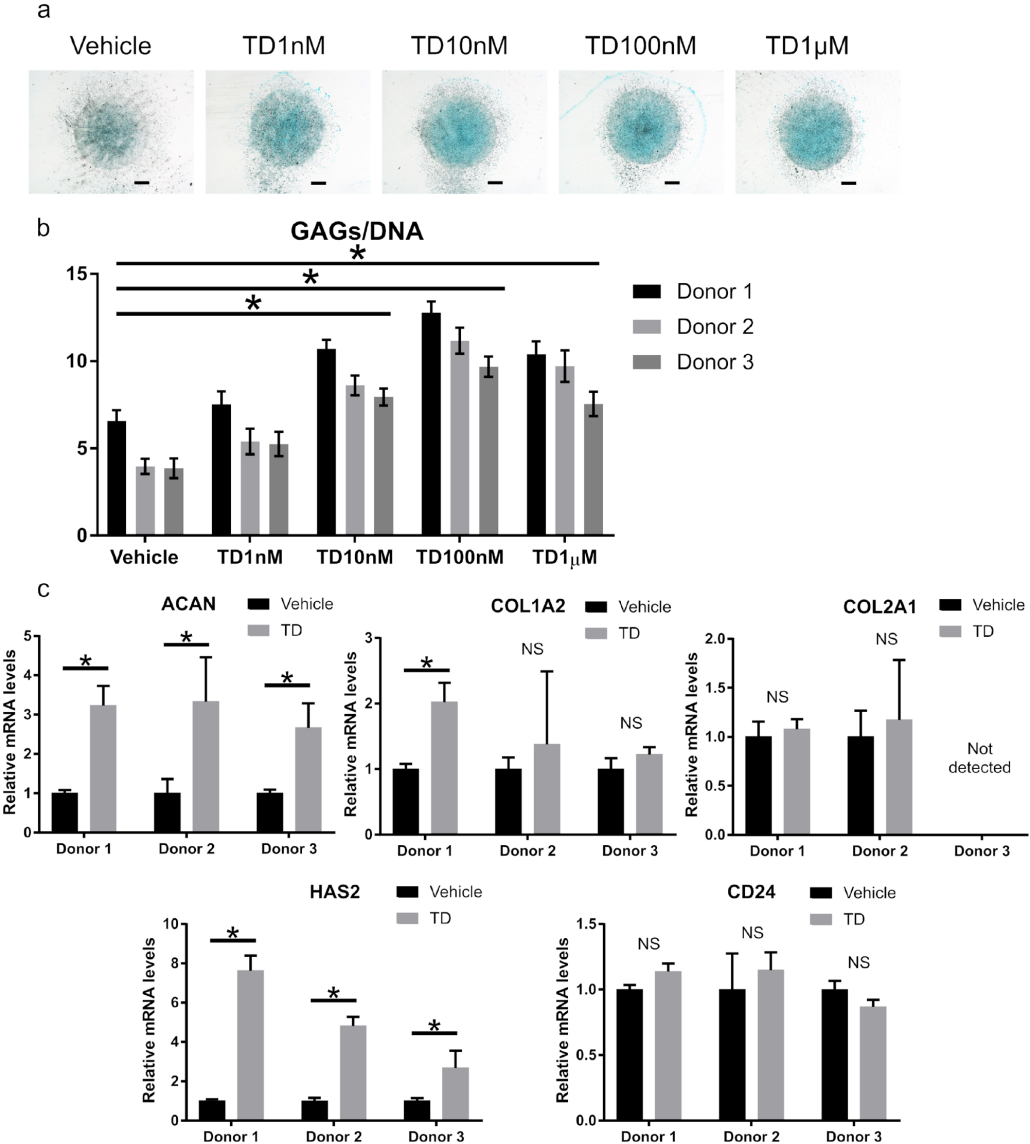
Two-dimensional micromass culture of human NP cells. (**a**) Alcian blue staining of human NP cells cultured with the vehicle or TD (1 nM to 1 μM) for 7 days (bar = 1,000 μm, representative data for Donor 1). (**b**) Sulfated GAG quantification of human NP cells cultured with the vehicle or TD (1 nM to 1 μM) for 7 days. Data represent mean ± S.D., n = 4 for each group, *; p < 0.05 by one-way ANOVA followed by the Dunnett test. (**c**) Real-time PCR analysis of human NP cells cultured with vehicle or TD (10 nM) for 7 days. Data represent mean ± S.D., n = 4 for each group, *; p < 0.05, *NS* not significant by Student’s t test. *NP* nucleus pulposus, *TD* TD-198946, *GAG* glycosaminoglycan.

We examined the effects of TD-198946 on hNPCs for future clinical applications. Consistent with the results in mNPCs, TD-198946 enhanced GAG synthesis in human NP cells (the highest effect achieved with 100 nM) (Fig. 2a,b). Although TD-198946 (10 nM) increased the expression of ACAN and HAS2, it did not affect the expression of COL2A1 and CD24 (Fig. 2c). Thus, we revealed the effects of TD-198946 on hNPCs for GAG synthesis.

### Phosphoinositide 3-kinase (PI3K)/Akt signaling pathway was involved in the effect of TD-198946

To elucidate the mechanism of action of TD-198946, we performed an mRNA sequencing assay to search the possible intracellular signaling pathways that were most affected by the administration of TD-198946. Most differentially expressed genes (DEGs) identified by Kyoto Encyclopedia of Genes and Genomes (KEGG) pathway analysis were involved in the PI3K/Akt signaling pathway, which was the most altered pathway by TD-198946 (Supplementary Fig. 1). To further evaluate the mechanism of action, we performed western blotting and Akt inhibitor analysis using three donor samples, one of which was obtained after RNA-seq analysis.

**Figure 3:**
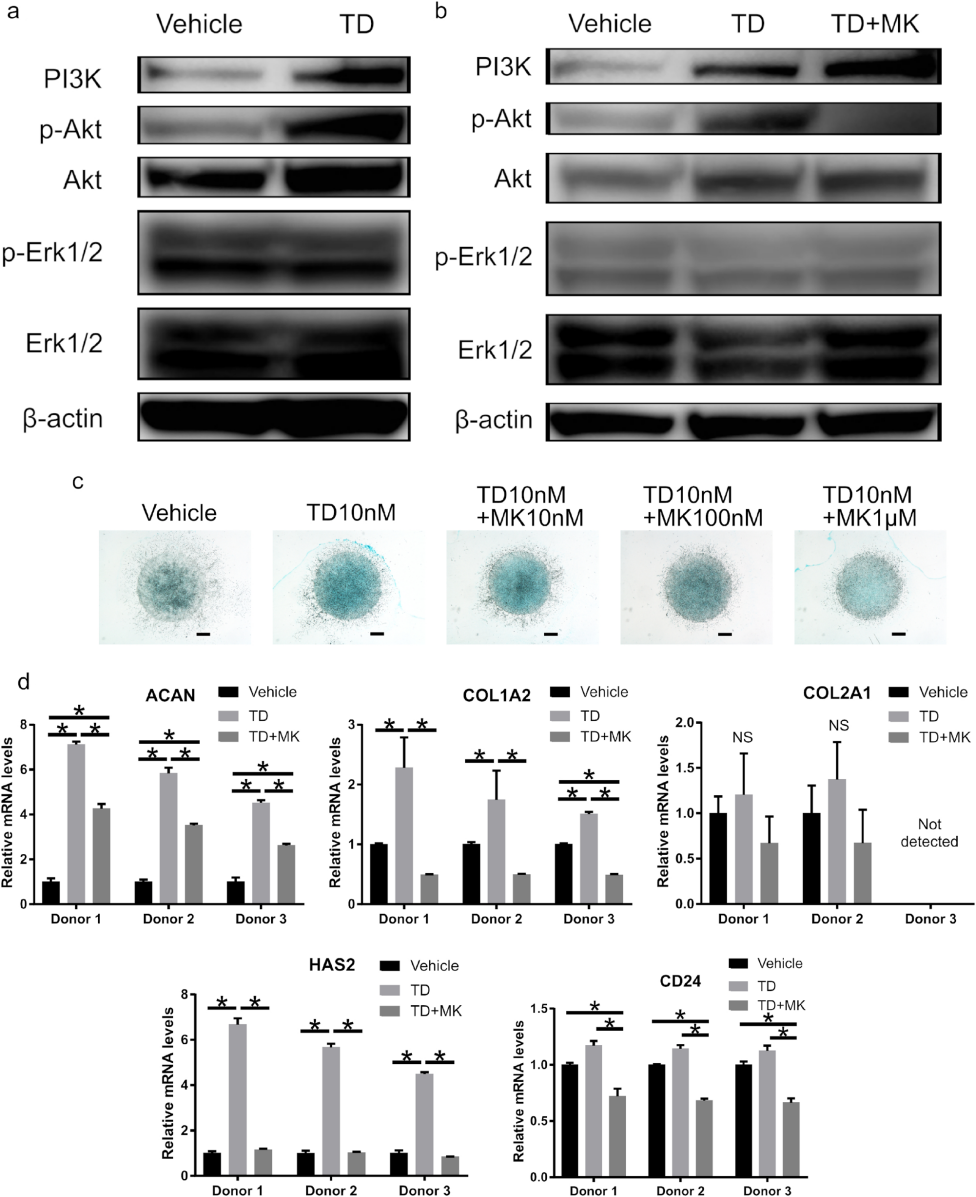
Involvement of TD-198946 in PI3K/Akt signaling. (**a**) Western blot assay of the hNPCs cultured with the vehicle or TD (10 nM). Representative data for Donor 1. (**b**) Western blot assay of the hNPCs cultured with vehicle, TD (10 nM), or TD (10 nM) with MK (100 nM). Representative data for Donor 1. (**c**) Alcian blue staining of human NP cells cultured with the vehicle, TD (10 nM), or TD (10 nM) with 10 nM to 1 μM of MK for 7 days (bar = 1,000 μm). Representative data for Donor 1. (**d**) Real-time PCR analysis of human NP cells cultured with the vehicle, TD (10 nM), or TD (10 nM) with MK (100 nM) for 7 days. Data represent mean ± S.D., n = 4 for each group, *; p < 0.05, *NS* not significant by one-way ANOVA followed by the Bonferroni test. *PI3K* phosphoinositide 3-kinase, *hNPCs* human nucleus pulposus cells, *TD* TD-198946, *MK* MK2206.

In western blotting, the expression levels of PI3K and phospho-Akt were increased by the administration of TD-198946, whereas the expression level of phospho-Erk1/2 was not affected (Fig. 3a, Supplementary Fig. 2).

We proceeded to determine whether the inhibition of PI3K/Akt signaling attenuates the effects of TD-19894 using the Akt inhibitor, MK-2206. A previous study reported that significant cell toxicity owing to MK-2206 occurs at concentrations ≥ 5 μM^16^. Thus, we added 10 nM–1 μM of MK-2206 to the TD-198946 (10 nM) supplemented medium. Akt was successfully inhibited by the decreased expression of phospho-Akt in the hNPCs cultured with TD-198946 (10 nM) and MK-2206 (100 nM) (Fig. 3b, Supplementary Fig. 3). The enhanced synthesis of GAG by TD was dose-dependently decreased by MK-2206 (Fig. 3c). Further, the increased expression of ACAN and HAS2 by TD-198946 (10 nM) was decreased by the addition of MK-2206 (100 nM) (Fig. 3d). There was no significant difference in the expression of COL2A1 between groups. Collectively, these findings indicate that the PI3K/Akt signaling pathway is involved in the effect of TD-198946.

### TD-198946 treated the immediate puncture-induced IDD in vivo

**Figure 4:**
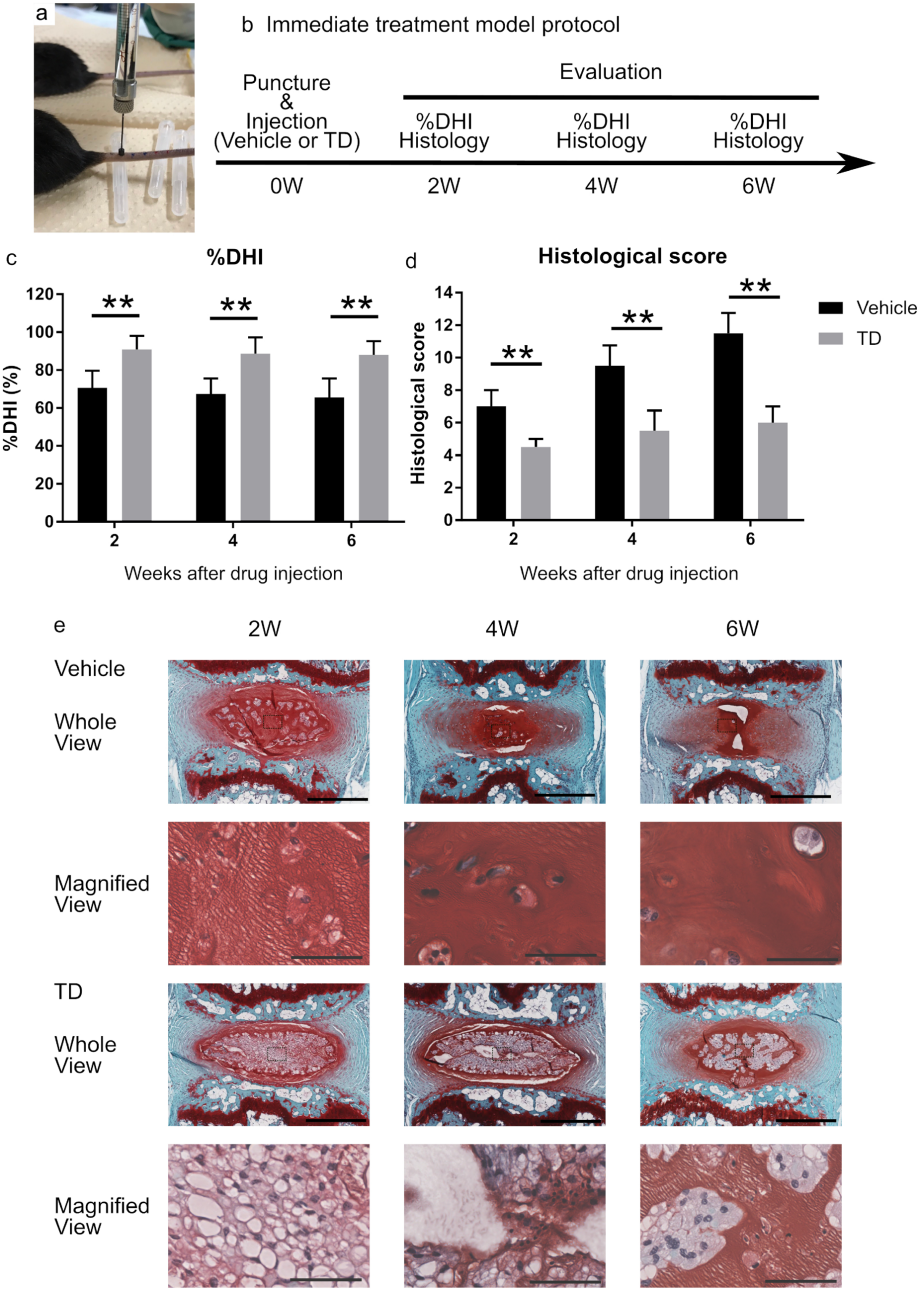
Immediate treatment model. (**a**) Injection of 5 μL of either vehicle or TD (100 nM). (**b**) Schedule of the prevention model protocol. (**c**) %DHI of either the vehicle or TD (100 nM) treated group. Data represent mean ± S.D., n = 8 for each group, **; p < 0.01 by Student’s t test. (**d**) Histological score of either the vehicle or TD (100 nM) treated group. Data represent median ± quartile deviation, n = 8 for each group, **; p < 0.01 by Mann–Whitney U test. (**e**) Safranin O staining of the intervertebral discs from either the vehicle or TD (100 nM) treated group (Whole View: bar = 500 μm, Magnified View: bar = 60 μm). *TD* TD-198946, *DHI* disc height index.

We examined whether TD-198946 could treat the immediate occurrence of IDD using a mouse tail-puncture induced IDD model (Fig. 4a,b). TD-198946 attenuated the loss in tail-puncture-induced disc height as measured by the % disc height index (DHI) at every time point (Fig. 4c, Supplementary Fig. 4a). The histology of the vehicle group highlighted the honeycomb appearance of the NP owing to the loss of NP cells at 2 weeks (Fig. 4e, Supplementary Fig. 5). Most NP cells disappeared until 4 weeks after the puncture. Six weeks after the puncture, NP tissue was lost and later replaced with fibrous tissue. However, in the TD-198946 group, the NP structures were well maintained, with abundant ECM until 6 weeks after drug injection; this occurred despite the decrease in NP cells with time. The histological score in the TD-198946 group was higher than that in the vehicle group at every time point (Fig. 4d). These findings suggest that TD-198946 prevented the occurrence of IDD in this prevention model.

### TD-198946 attenuated the progression of mild IDD in vivo

**Figure 5:**
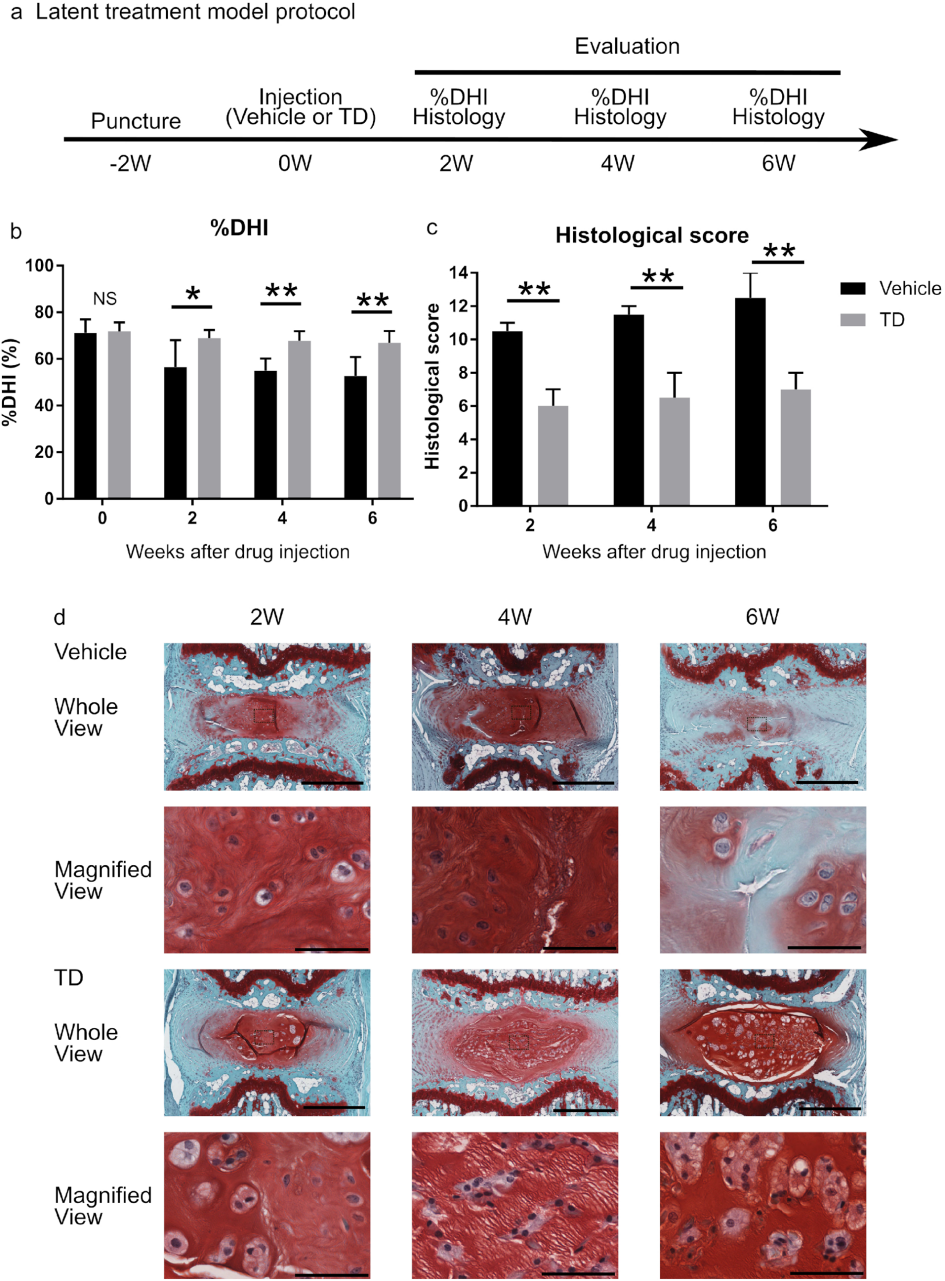
Latent treatment model. (**a**) Schedule of the treatment model protocol. (**b**) %DHI of either the vehicle or TD (100 nM) treated group. Data represent mean ± S.D., n = 6 for each group, *; p < 0.05, **; p < 0.01, NS; not significant by the Student’s t test. (**c**) Histological score of either the vehicle or TD (100 nM) treated group. Data represent median ± quartile deviation, n = 6 for each group, **; p < 0.01 by Mann–Whitney U test. (**d**) Safranin O staining of the intervertebral discs from either the vehicle or TD (100 nM) treated group (Whole View: bar = 500 μm, Magnified View: bar = 60 μm). *TD* TD-198946, *DHI* disc height index.

We sought to determine whether TD-198946 could treat mild IDD by employing a mild mouse IDD model. Briefly, the tail of the mouse was punctured 2 weeks before TD-198946 injection (Fig. 5a). In the vehicle group, the %DHI decreased over time during the 6-week observation period. However, in the TD-198946 group, the %DHI was maintained for 6 weeks. Additionally, this group had a higher %DHI than the vehicle group after injection at every time point (Fig. 5b, Supplementary Fig. 4b). The TD-198946 group had a higher histological score at every time point than the vehicle group (Fig. 5c). In the vehicle group, the number of NP cells significantly decreased at 2 weeks (4 weeks after puncture) and there were no NP cells at 6 weeks (8 weeks after puncture) (Fig. 5d, Supplementary Fig. 6). In the TD-198946 group, the number of NP cells in the NP decreased; however, they remained inside the ECM until 6 weeks. These findings suggest that TD-198946 could attenuate the progression of mild IDD.

## Discussion

In this study, we demonstrated that TD-198946 enhanced the production of GAG in NP cells via the PI3K/AKT signaling pathway. Furthermore, administering TD-198946 into the intervertebral disc of a mouse model could attenuate the progression of IDD induced by needle puncture.

Our previous studies revealed that TD-198946 enhances GAG synthesis in mouse chondrocytes, human bone marrow stromal cells, and human synovium-derived stem cells (hSSCs)^10,17,18^. Moreover, our previous study identified a unique effect of TD-198946 on hyaluronan secretion by hSSCs^17^. Consistent with previous studies, TD-198946 was found to enhance the gene expression of aggrecan and hyaluronan synthase 2, resulting in increased GAG synthesis in both mNPCs and hNPCs of all donors. Therefore, we concluded that the main effect of TD-198946 was the enhancement of the production of GAG. This increased production of GAGs might play a significant role in the preservation of disc height in the mouse IDD model. Chondrocytes can synthesize GAGs and type 2 collagen; however, the ECM of NP can be distinguished from that of cartilage by the predominance of GAG in the ECM of NP. Furthermore, despite the 3:1 ratio of GAGs:hydroxyproline in juvenile human cartilage, which decreases to 2:1 with age, a much higher ratio is found at all ages in hNPCs. The ratio of juvenile hNPCs is 25:1, whereas that of young adult hNPCs is 27:1; the latter, however, decreases to 5:1 with age. The increase in GAG synthesis in NP cells owing to TD-198946 is favorable for the predominant GAG feature in the ECM of NP.

The effect of TD-198946 on the expression of COL2A1 in human NP cells was not significant. One possible explanation is the difference in degeneration between mNPCs and hNPCs. The mNPCs were isolated from young, healthy NP tissue under a microscope, whereas the hNPCs were isolated from excised NP tissue during lumbar disc herniation surgery. The hNPCs from surgically treated patients for disc disease in this study were considered to be more degenerated than mNPCs, and the degree of degeneration should be different among human donors. Another explanation is that the increase in the expression of Col2a1 by TD-198946 was milder than that of Acan or Has2 even in mNPCs. Moreover, our previous study using hSSCs also revealed that TD-198946 enhances GAG production but does not affect the expression of COL2A1 in hSSCs^17^. Thus, we considered that the expression of COL2A1 is not a direct target of TD-198946. TD-198946 synergistically increases the expression of COL2A1 when combined with transforming growth factor-β3 or bone morphogenetic protein-2^17,19^. Further research is needed to explore the optimal combinations of TD-198946 and growth factors that enhance the expression of COL2A1 in hNPCs.

**Figure 6:**
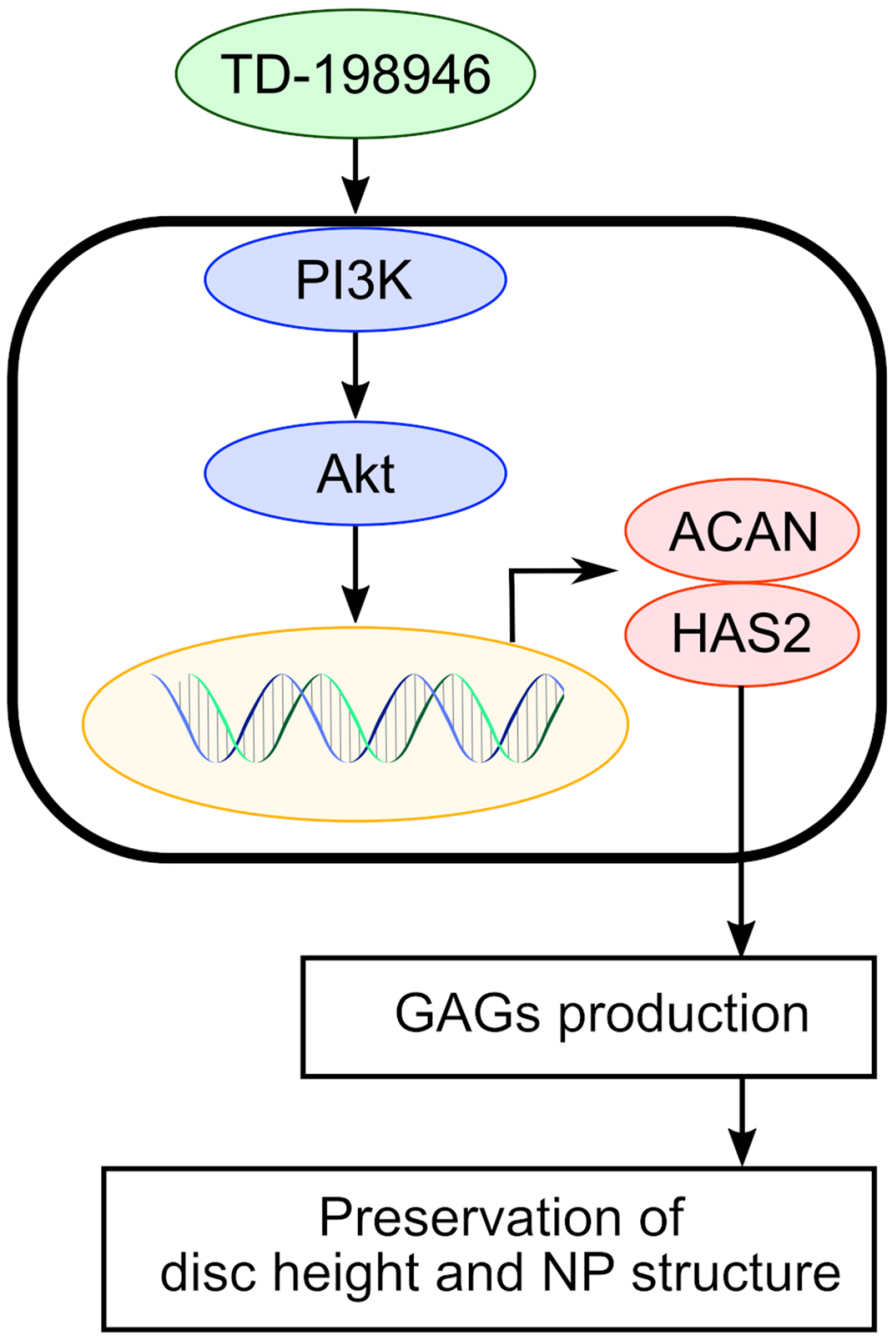
The effect of TD-198946 on NP cells. The administration of TD-198946 activated the PI3K/Akt signaling pathway and increased the expression of Acan and Has2 in NP cells. Thus, the increased GAG production from NP cells preserved the intervertebral disc height and NP structure. *TD* TD-198946, *GAG* glycosaminoglycan, *NP* nucleus pulposus, *PI3K* phosphoinositide 3-kinase.

This study indicates that the PI3K/Akt signaling pathway is involved in the effect of TD-198946, although further research is needed to elucidate the precise mechanism of action by TD-198946. The activation of the PI3K/Akt pathway has been reported to protect against IDD through different mechanisms, such as the increase in ECM content, prevention of apoptosis, facilitation of cell proliferation, induction or prevention of autophagy, alleviation of oxidative damage, and adaptation to a hypoxic microenvironment^20–23^. The activation of the PI3K/Akt signaling may affect ECM content by enhancing the transcription of aggrecan in rat NP cells^24^. In this study, the administration of TD-198946 enhanced the PI3K/Akt signaling pathway in NP cells and increased the expression of aggrecan and hyaluronan synthase 2. After the puncture of the disc, the number of NP cells decreased following treatment with TD-198946. However, the increased production of GAG in NP cells was assumed to contribute to the maintenance of the intervertebral disc height and NP structure (Fig. 6).

This study had several limitations. First, only a few human donors were enrolled, most of whom were young. Therefore, further research with more human donors is needed to evaluate the efficacy of TD-198946 in NP cells of older generations. Second, the hNPCs in this study were obtained surgically and were more degenerated than mNPCs. The inconsistency of the response to TD-198946 may be attributable to these differences. However, the increased expression of ACAN and HAS and the enhanced GAG synthesis in hNPCs of all donors by TD-198946 suggest that TD-198946 is a candidate therapeutic molecule for IDD. Third, the length of time spent by TD-198946 inside the intervertebral disc space after intra-disc injection was not measured. Because frequent intra-disc injection might accelerate IDD, only one intra-disc injection was administered. Further, as the fibrous annulus fibrosus and hypoperfusion surround NP owing to the avascular, TD-198946 was expected to remain in the space for a certain amount of time. A further study is thus required to trace the length of time spent by TD-198946 inside the disc space. Finally, the biomechanical environment of the mouse coccygeal vertebrae differs from that of the human spine. Thus, a pre-clinical animal model whose spine has a similar biomechanical environment to the human spine is desired for future clinical application.

In conclusion, the small compound, TD-198946, increased the ability of NP cells to produce GAGs via the PI3K/Akt signaling pathway and protected against early IDD.

## Methods

### Reagents

Herein, the same lot of TD-198946 employed in our previous study^17^ was utilized for all experiments. TD-198946 was dissolved in dimethyl sulfoxide (DMSO, Sigma-Aldrich, St. Louis, MO) and diluted in phosphate-buffered saline (PBS) (1:1,000) for the in vivo analysis, and culture medium (1:1,000) for the in vitro analysis. DMSO diluted in PBS (1:1,000) and the culture medium (1:1,000) were respectively used as the vehicle in the in vivo and in vitro analyses.

### Isolation of mouse NP cells and human NP cells

Male C57BL6J mice (age, 10–12 weeks) were purchased from Oriental Yeast Co., Ltd. (Tokyo, Japan). mNPCs were cultured as described previously^25^. With this method, NP cells from five mice were required to prepare one set of NP cells to perform in vitro experiments. Briefly, mouse NP tissues were digested with 0.1% pronase (Roche, Indianapolis, IN) at 37 °C for 30 min and 0.2% collagenase type 2 (Worthington, Lakewood, NJ) at 37 °C for 2 h. After digestion, the mNPCs were cultured in 3D collagen gel (Cellmatrix type I-A, Nitta Gelatin, Osaka, Japan) in Dulbecco’s Modified Eagle’s Medium (DMEM, Nacalai Tesque, Kyoto, Japan) with 10% fetal bovine serum (FBS, Thermo Fisher Scientific, Waltham, MA) and 1% antibiotic/antimycotic solution (A/A, Sigma-Aldrich) under a 5% CO_2_ and 5% oxygen atmosphere at 37 °C. After primary 3D collagen gel culture, the gel was digested, and the mNPCs were cultured in a 2D monolayer in expansion medium (DMEM with 10% FBS and 1% A/A, with 10 ng/mL of bFGF [recombinant human basic fibroblast growth factor, Fujifilm Wako, Osaka, Japan]). Low-passage cells (passage 2) were used for all experiments. We prepared three sets of NP cells from 15 mice, and all experiments were performed using each set of NP cells.

After the retrieval of informed consent from patients, human NP tissue was isolated from three donors during nucleotomy surgery for lumbar intervertebral disc herniation. The gender, age, and disc levels of the donors are listed in Supplementary Table 1. The tissues were digested with 0.1% pronase at 37 °C for 30 min and 0.2% collagenase type 2 at 37 °C overnight. After digestion, the isolated hNPCs were cultured as a monolayer in expansion medium under a 5% CO_2_ and 5% oxygen atmosphere at 37 °C. Low-passage cells (passage 1) were used for all experiments.

### Two-dimensional micromass culture

Two-dimensional micromass culture was performed as described previously^17,25^. Briefly, a high concentration of mNPCs or hNPCs was cultured in micromass (1 × 10^5^ cells/10 μL) in chondrogenic basal medium (DMEM with 1% ITS [insulin, transferrin, selenium, Corning Inc., Corning, NY], 50 μg/mL ascorbic acid [Sigma-Aldrich], 40 μg/mL L-proline [Fujifilm Wako], 1% FBS, and 1% A/A) supplemented with vehicle or TD-198946 (1 nM to 1 μM) for 7 days. The medium was changed twice per week.

### Alcian blue staining

Both mNPCs and hNPCs were fixed with 4% paraformaldehyde, stained with alcian blue (pH 1.0) for 3 h, and washed with distilled water.

### Sulfated GAG quantification

The micromass of the mNPCs or the hNPCs was first digested with 0.05% papain (Sigma‐Aldrich) for 3 h at 65 °C with shaking. Thereafter, the sulfated GAG content was measured by a dimethylmethylene blue dye‐binding assay (Blyscan Glycosaminoglycan Assay Kit, Biocolor, Westbury, NY) with a kit containing chondroitin sulfate as the standard. Cellularity was measured according to the double‐stranded DNA (dsDNA) content using a Qubit 3.0 Fluorometer (Thermo Fisher Scientific) and the Qubit dsDNA HS Assay kit (Thermo Fisher Scientific).

### Real-time PCR assay

The mNPCs or hNPCs were homogenized in TRIzol Reagent (Invitrogen, Carlsbad, CA). Total RNA was extracted using the Direct-zol RNA kit (Zymo Research, Orange, CA). Then, the total RNA was converted to cDNA using ReverTra Ace qPCR RT Master Mix (Toyobo, Osaka, Japan). Gene expression was measured using quantitative real-time PCR with SYBR green master mix (Thermo Fisher Scientific) for mNPCs and TaqMan Fast Advanced Master Mix (Thermo Fisher Scientific) for hNPCs on the Step One Plus Real-Time PCR System (Applied Biosystems, Foster City, CA). The expression of the ECM synthesis genes (Acan, Has2, Col1a1, and Col2a1), the NP-specific marker (Cd24)^5,13–15^, and the endogenous gene (Gapdh) was evaluated. The primer sequences used for real-time PCR are listed in Supplementary Tables 2 and 3. The mRNA levels were calculated using the relative quantitation standard curve method and normalized to the level of Gapdh in each sample.

### RNA sequencing and pathway analysis

After total RNA was extracted from hNPCs (Donors 1 and 2) cultured with the vehicle or TD-198946 (10 nM) for 7 days, an mRNA sequencing analysis was performed at BGI Tech Solutions Co., Ltd. (Hong Kong) using the BGISEQ-500 platform. A transcript library was constructed for all samples, and all transcripts were sequenced with the 50-bp single-end sequencing technology. The differential analysis compared the differential transcripts between the control and the TD-198946 group. DEGs between the groups were detected with DEseq2 by BGI Tech Solutions. DEseq2 is based on the negative binomial distribution performed as described previously^26^. The false discovery rate (FDR) for each P value was calculated. Generally, the terms with an FDR less than 0.01 were defined as significantly enriched. The P value cut-off was set at 0.05. A fold change ≥ 2.00 or ≤ 0.50 and an adjusted P value ≤ 0.05 were considered to indicate significance. KEGG pathway analysis was performed to identify significant DEGs^27–30^. Using the KEGG annotation result, DEGs were classified according to the official classification to determine the number of DEGs in the most enriched pathway.

### Western blotting

The hNPCs were cultured with serum-free DMEM for 24 h. After serum starvation, the hNPCs were cultured with DMEM supplemented with the vehicle or TD-198946 (10 nM) for 60 min. Thereafter, the cells were washed with PBS and lysed in RIPA buffer (Nacalai Tesque) containing a protease/phosphatase inhibitor cocktail (Cell Signaling Technology, Danvers, MA). The total protein was sonicated, centrifuged at 12,000 rpm for 5 min at 4 °C, and quantified using the Pierce Rapid Gold BCA Protein Assay Kit (Thermo Fisher Scientific). Equal amounts of protein were separated on Bolt Bis–Tris Plus, 4–12% precast polyacrylamide gels (Thermo Fisher Scientific). Fractionated proteins were transferred onto a PVDF membrane using the Mini Blot Module (Thermo Fisher Scientific). After 1 h of blocking with Phospho-blocker (Cell Biolabs Inc., San Diego, CA), the membrane was probed overnight at 4 °C with a primary antibody followed by 1 h at room temperature with anti-rabbit IgG HRP-linked antibody (7,074, Cell Signaling Technology). Immunodetection was performed with the Amersham ECL Prime western blotting detection reagent (GE Healthcare, UK). The membrane was visualized using MF-ChemiBIS 3.2 (DNR Bio-Imaging Systems Ltd., Israel). The primary antibodies used for western blot analysis are listed in Supplementary Table 4. The bands were quantified using Image J software (National Institutes of Health, Bethesda, MD). β-Actin was used as the loading control for internal correction.

### Akt inhibitor analysis

The Akt-inhibitor, MK-2206 (Chemscene, Monmouth Junction, NJ), was used. The hNPCs were cultured in micromass in chondrogenic basal medium supplemented with TD-198946 (10 nM) and MK-2206 (10 nM to 1 μM) for seven days for alcian blue staining and real-time PCR. The hNPCs were also cultured with DMEM supplemented with TD-198946 (10 nM) and MK-2206 (100 nM) (TD + MK2206) for 60 min for western blot analysis.

### Mice

Male C57BL6J mice (age, 10–12 weeks) were purchased from Oriental Yeast Co., LTD. (Tokyo, Japan) and maintained under standard animal housing conditions (12 h light–12 h dark cycle and free access to food and water). All mice were maintained under specific-pathogen-free conditions and handled according to the guidelines of the Institutional Animal Care and Use Committee of Osaka University Graduate School of Medicine.

### IDD animal model

The mouse IDD model by AF puncture using a 33G needle has been established^7,31^. The AF puncture using a 33G needle can lead to a mild IDD. Thus, the 33G needle was recommended for the study of intervertebral disc regeneration. Briefly, under general anesthesia, the segments of tail intervertebral discs were shown by high-resolution micro-computed tomography (CT) (Rigaku, Tokyo, Japan) at 90 kV and 160 mA. Micro CT was performed using a fixed metal marker on the tail side and skin marker at the puncture level, which was created based on the relative positional relation between the metal marker and the disc levels. Then, the discs (Co3-Co4 segment) were punctured percutaneously with a 33-gauge needle (Neuros Syringes, Hamilton, Reno, NV) through the annulus fibrosus to the depth of the needle stopper (1 mm) from the dorsal to the ventral side. Two experimental models (immediate and latent treatment models) were established according to the timing of the puncture and injection. In the immediate treatment model, 5 μL of either the vehicle or TD-198946 (100 nM) was injected into the disc using the Neuros Syringe at the same time as the tail disc puncture (n = 8 for each group, total puncture number = 1). In the latent treatment model, the drugs were injected into the disc using the Neuros Syringe at 2 weeks following the puncture (n = 6 for each group, total puncture number = 2). The injection was performed slowly over 1 min to prevent the acute increase in internal pressure of the disc. After the injection, the needle was held for another 1 min to promote perfusion of the solution into the tissue and prevent the leakage from the disc, as previously described^7,32^.

### Radiographic assessment of the disc height

A high-resolution micro-computed tomography was used to scan the tails at 90 kV and 160 mA. The DHI was measured using Image J software) using a previously reported method^33^. The disc height and two adjacent vertebral body heights were obtained as averages of the anterior, middle, and posterior portions. DHI was calculated by dividing disc height by the average of the two adjacent vertebral body heights and multiplying the result by 100. The change in DHI was expressed as %DHI (post-injection DHI/pre-injection DHI).

### Histological analysis

The intervertebral discs were fixed in 4% paraformaldehyde in PBS and decalcified with 20% EDTA until soft and pliable. Thereafter, the intervertebral discs were dehydrated using serial ethanol and cleared in xylene. After the samples were embedded in paraffin wax, sections were created at the central part of the intervertebral disc using the shape of the NP, AF, and epiphyseal plate as the indices. However, it was technically challenging to create histological images of all samples at precisely the same depth. Sections were cut 3-μm-thick and stained with hematoxylin and eosin (H&E) and Safranin-O fast green (SO) according to the standard protocol. IDD generated via the tail puncture method was quantified with a histological grading score as described previously^34^. Briefly, the grading score of IDD is based on the morphological features of the NP, AF, and NP/AF boundary, with higher scores representing higher levels of degenerative changes (from 0 [standard] to 14 [severely degenerated]).

### Statistical analysis

GraphPad Prism 7 (GraphPad Inc., La Jolla, CA) was used for the analysis. Differences in the measured variables between the groups were analyzed using the Student’s *t* test or Mann–Whitney U test, as appropriate. Differences in the measured variables between multiple groups were analyzed using one-way ANOVA followed by the Dunnett test or the Bonferroni test. Differences with a p value < 0.05 were considered significant.

### Ethics statement

The Ethical Review Board of Osaka University Hospital approved the experiments performed with human tissue (No. 19423). Written informed consent was obtained from each patient and/or from either the parent or the legally authorized representative of the patient under eighteen years old according to the principles of the Declaration of Helsinki and the laws and regulations of Japan. The Animal Experimental Committee of Osaka University Graduate School of Medicine approved all animal studies (No. 280038). All methods were performed per relevant laboratory guidelines and regulations.

## Supplementary information

Supplementary Information

## Data Availability

The datasets generated during and/or analyzed during the current study are available from the corresponding authors on reasonable request.
