# Supplementary material for: The small compound, TD-198946, protects against intervertebral degeneration by enhancing glycosaminoglycan synthesis in nucleus pulposus cells
Source: Sci Rep. 2020 Aug 25;10:14190. doi: 10.1038/s41598-020-71193-6 (PMC7447806; doi:10.1038/s41598-020-71193-6)
Supplement: Supplementary file 1 — Supplementary Information [file 41598_2020_71193_MOESM1_ESM.pdf]

The small compound, TD-198946, protects against intervertebral degeneration by enhancing glycosaminoglycan synthesis in nucleus pulposus cells

Junichi Kushioka, MD[a]; Takashi Kaito, MD, PhD[a*]; Ryota Chijimatsu, PhD[b]; Rintaro Okada, MD[a]; Hiroyuki Ishiguro, MD, PhD[a]; Zeynep Bal, MSc[a]; Joe Kodama, MD[a]; Fumiko Yano, PhD[b]; Taku Saito, MD, PhD[c]; Ung-il Chung, MD, PhD[d]; Sakae Tanaka, MD, PhD[c]; Hideki Yoshikawa, MD, PhD[a]

[a] Department of Orthopaedic Surgery, Osaka University Graduate School of Medicine, 2-2 Yamadaoka, Suita, Osaka 565-0871, Japan

[b] Bone and Cartilage Regenerative Medicine, Graduate School of Medicine, The University of Tokyo, 7-3-1 Hongo, Bunkyo-ku, Tokyo 113-8655, Japan

[c] Sensory and Motor System Medicine, Graduate School of Medicine, The University of Tokyo, 7-3-1, Hongo, Bunkyo-ku, Tokyo 113-8655, Japan

[d] Center for Disease Biology and Integrative Medicine, The University of Tokyo, 7-3-1, Hongo, Bunkyo-ku, Tokyo 113-8655, Japan

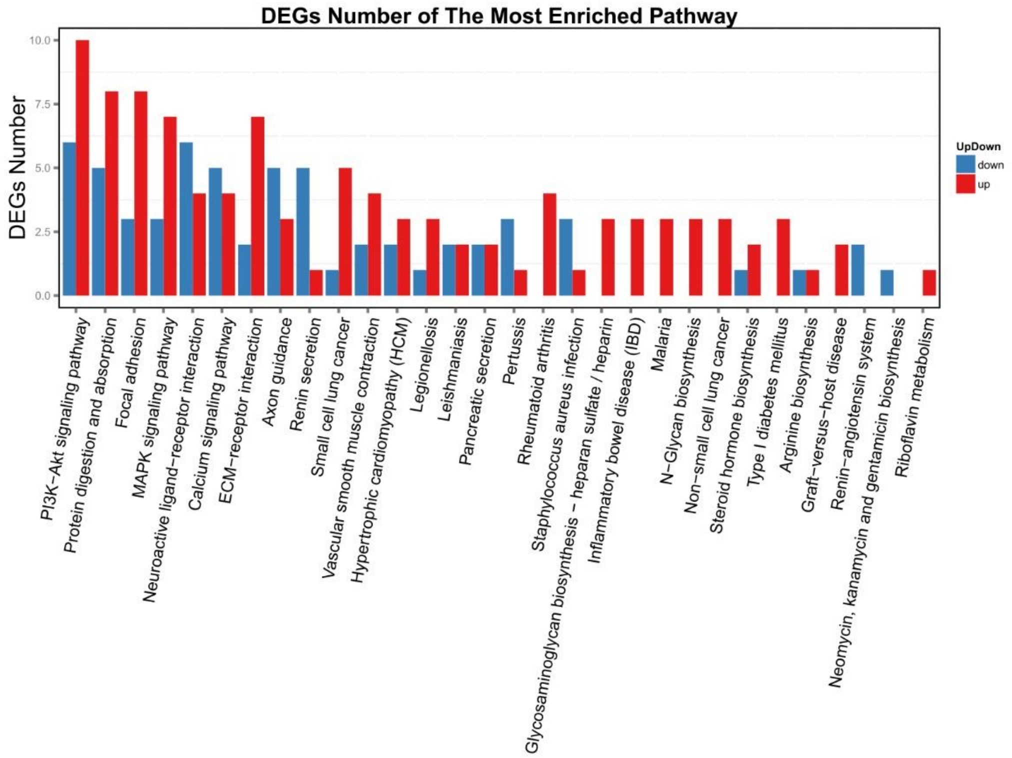

Supplementary Figure 1.

KEGG pathway analysis results for the number of DEGs in the most enriched pathway between hNPCs (Donor 1 and 2) cultured with the vehicle or TD (10 nM) for seven days. KEGG; Kyoto Encyclopedia of Genes and Genomes, DEGs; Differentially expressed genes

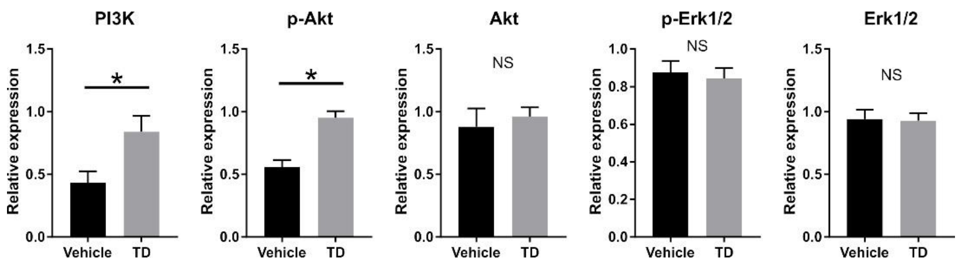

Supplementary Figure 2.

Quantitative western blot of the hNPCs cultured with the vehicle or TD (10 nM).

Data represent mean ± S.D., n=3 for each group, *; $p < 0.05$, NS; not significant by Student's t test. TD; TD-198946

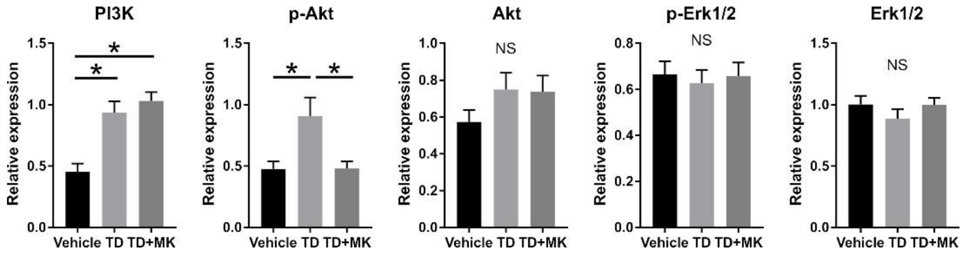

Supplementary Figure 3.

Quantitative western blot of the hNPCs cultured with vehicle, TD (10 nM), or TD (10 nM) with MK (100 nM).

Data represent mean ± S.D., n=3 for each group, *; $p < 0.05$, NS; not significant by one-way ANOVA followed by the Bonferroni test. TD; TD-198946, MK; MK2206

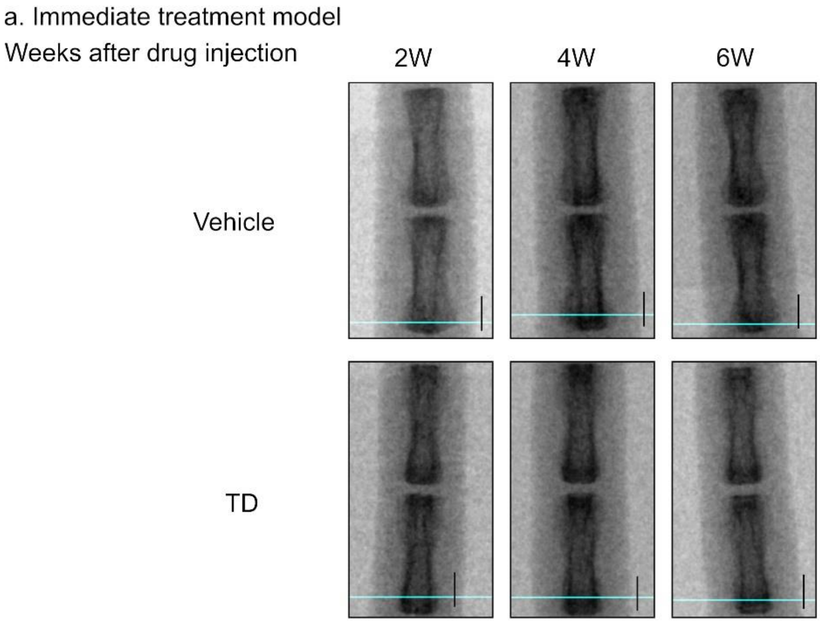

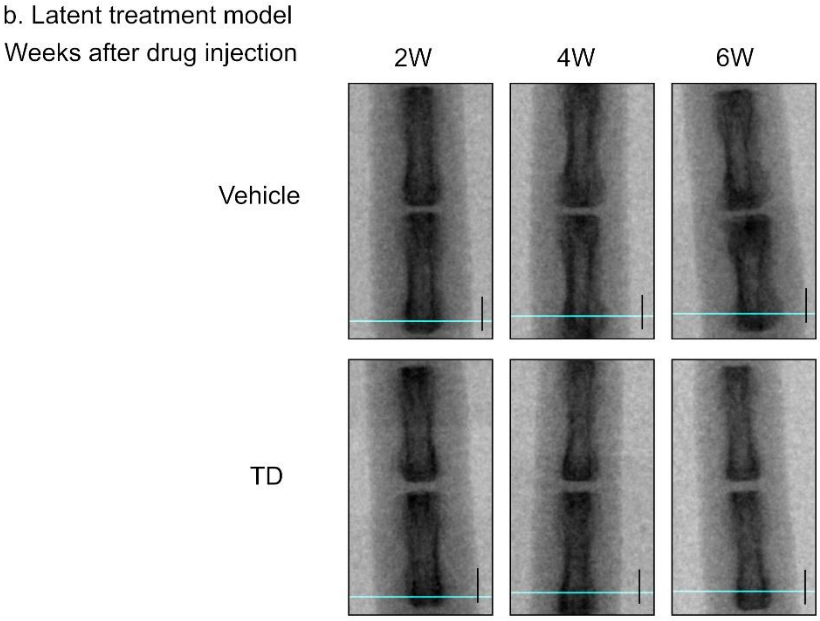

Supplementary Figure 4.

(a) Radiographic assessment of disc height in the immediate treatment model.

(b) Radiographic assessment of disc height in the latent treatment model.

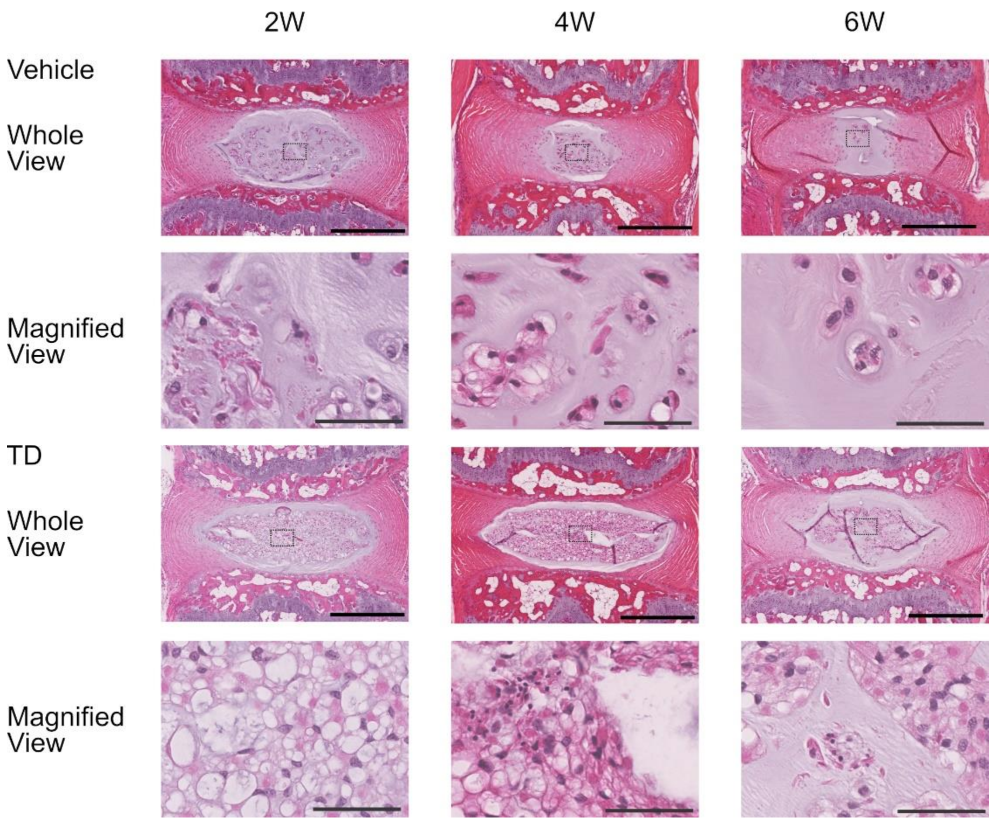

Supplementary Figure 5. Immediate treatment model.

Hematoxylin and eosin staining of the intervertebral discs from either the vehicle or TD (100 nM) treated group (Whole View: bar=500 μm, Magnified View: bar=60 μm).

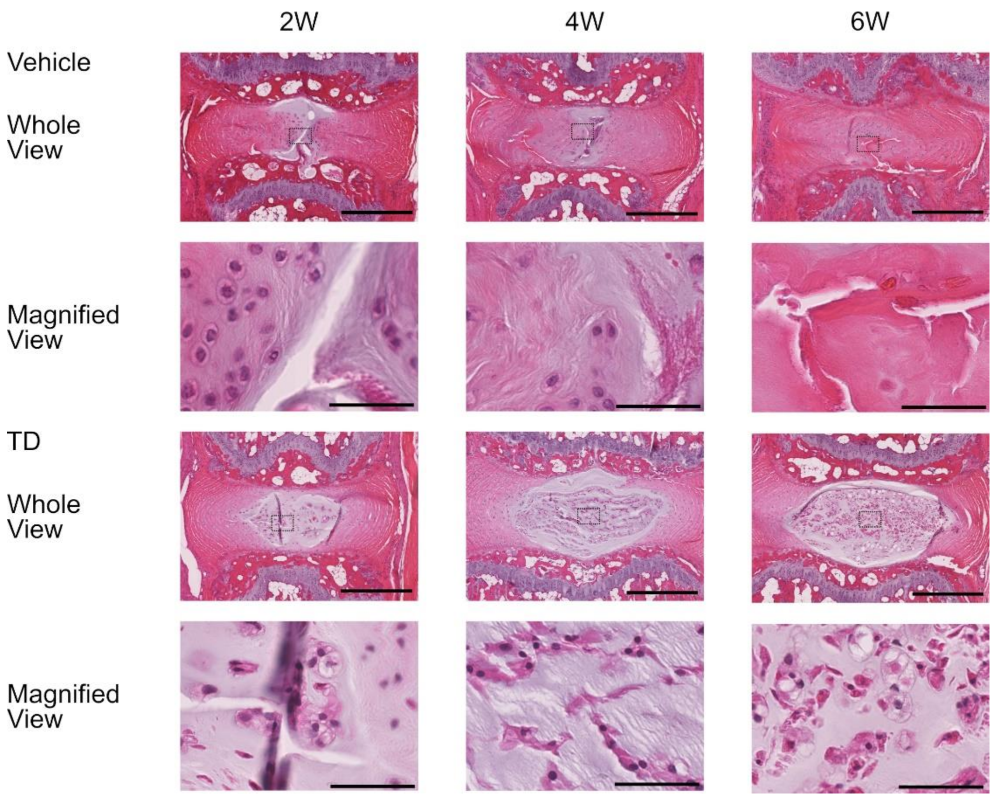

Supplementary Figure 6. Latent treatment model.

Hematoxylin and eosin staining of the intervertebral discs from either the vehicle or TD (100 nM) treated group (Whole View: bar=500 μm, Magnified View: bar=60 μm).

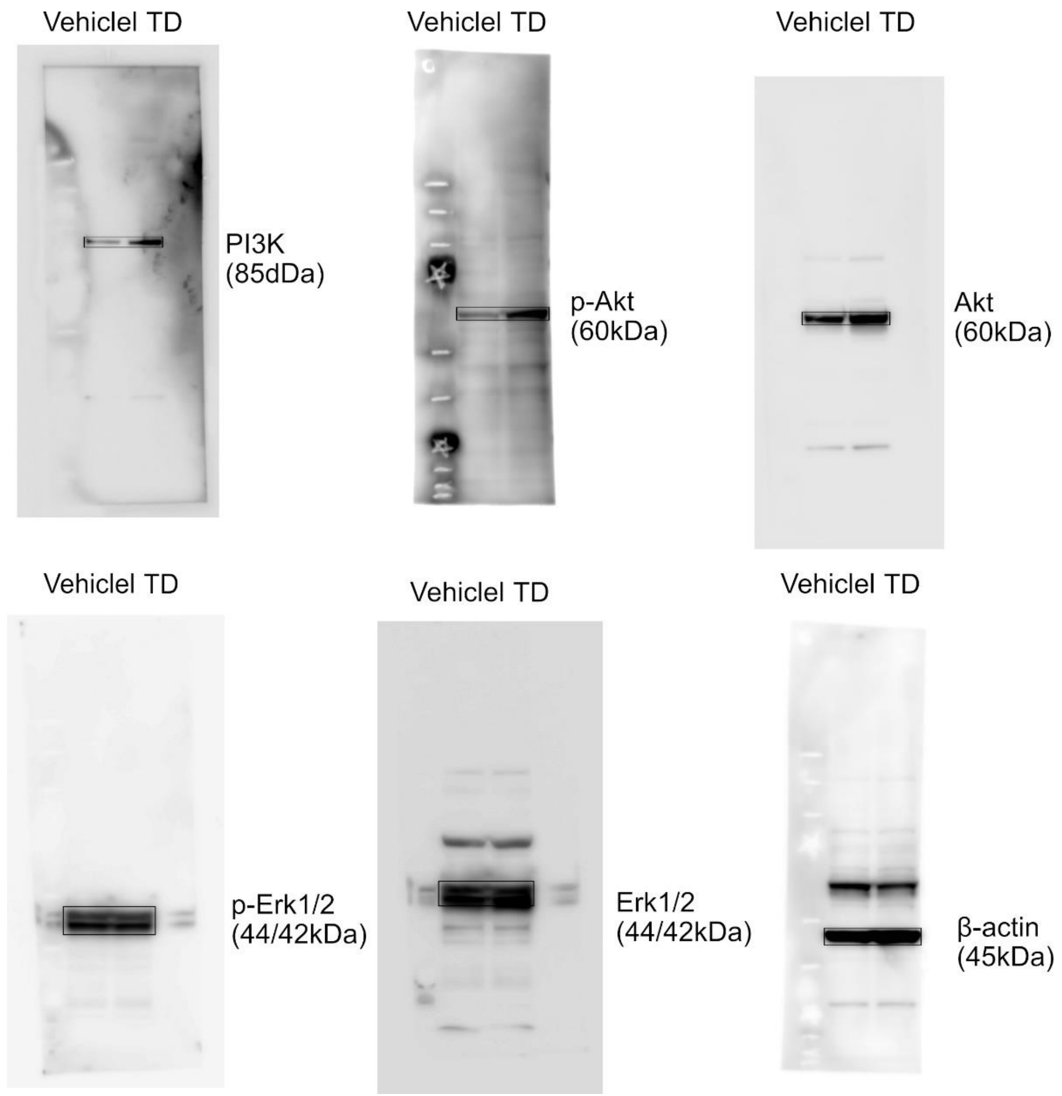

Supplementary Figure 7.

Western blot assay of the hNPCs cultured with the vehicle or TD (10 nM). Representative data for Donor 1.

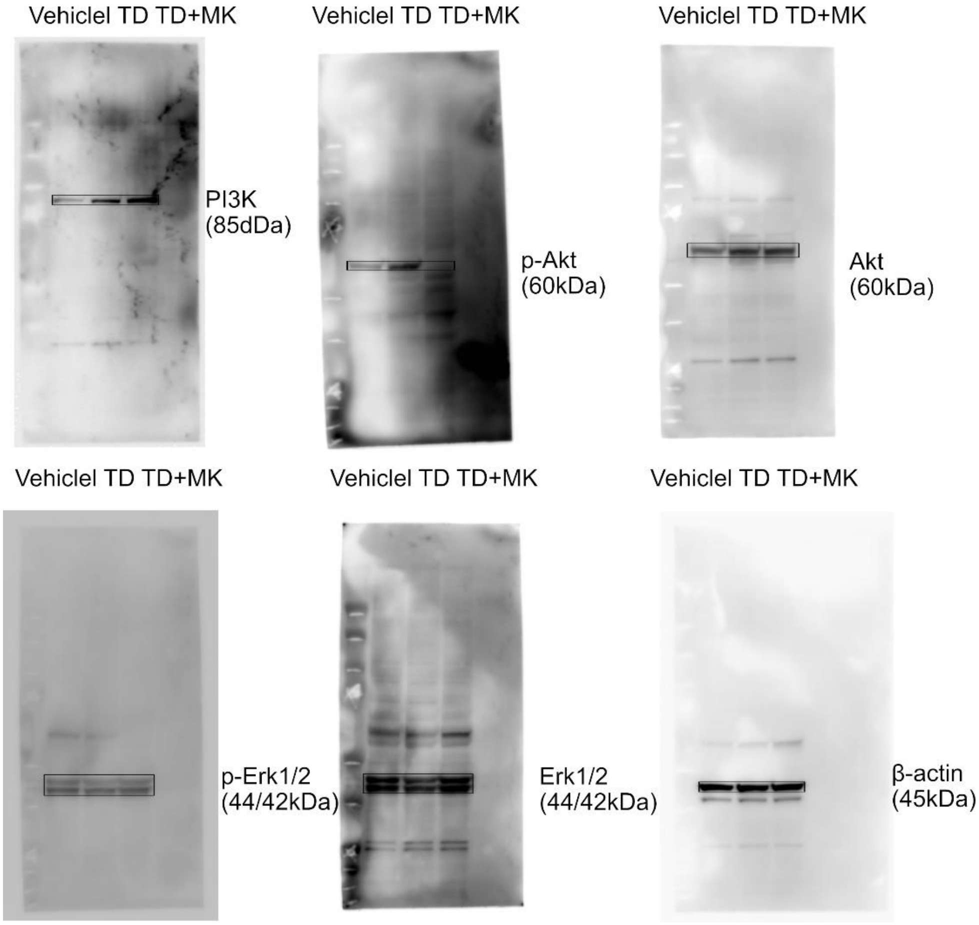

Supplementary Figure 8.

Western blot assay of the hNPCs cultured with vehicle, TD (10 nM), or TD (10 nM) with MK (100 nM). Representative data for Donor 1.

Supplementary Table 1. Donor information of intervertebral disc herniation

Donor No.	Sex	Age	Disc levels
1	Female	21	L5/S
2	Male	19	L4/5
3	Female	16	L4/5

Supplementary Table 2. Primer sequences for gene expression analysis using SYBR green master mix

Gene	Forward	Reverse
GAPDH	GGGTGTGAACCACGAGAAAT	ACTGTGGTCATGAGCCCTTC
Acan	AACAACTGCAGGCTGCCTAT	CCAGGGAACTCGTCCTTGTC
Col1a1	CTGACGCATGGCCAAGAAGA	ATACCTCGGGTTTCCACGTC
Col2a1	GGTGTGAAGGGTGAGAGTGG	CCTGGCTGGCCATCGTTAC
Has2	CGGTCCAAGTGCCTTACTGA	AGCCACTCTCGGAAGTAGGA
Cd24	TCACTCACCACAAGCACCAA	GCCAAGGCCAACATGATTCTG

Supplementary Table 3. Taqman assay list

Gene symbol	Assay code
GAPDH	Hs02758991_g1
COL1A2	Hs00164099_m1
COL2A1	Hs00264051_m1
ACAN	Hs00153936_m1
HAS2	Hs00193435_m1
CD24	Hs02379687_s1

Supplementary Table 4. Antibodies used in western blotting

Target Antigen	Company	Catalog No.	Dilution
PI3K	Cell Signaling Technology (Danvers, MA)	4257	1/1000
Phospho-Akt	Cell Signaling Technology	4058	1/1000
Akt	Cell Signaling Technology	9272	1/1000
Phospho-Erk1/2	Cell Signaling Technology	9101	1/1000
Erk1/2	Cell Signaling Technology	4695	1/1000
β-actin	Cell Signaling Technology	4970	1/1000
